# Aluminium matrix tungsten aluminide and tungsten reinforced composites by solid-state diffusion mechanism

**DOI:** 10.1038/s41598-017-12302-w

**Published:** 2017-09-28

**Authors:** Hanzhu Zhang, Peizhong Feng, Farid Akhtar

**Affiliations:** 1 Division of Materials Science, Luleå University of Technology, 971 87 Luleå, Sweden; 2 School of Materials Science and Engineering, China University of Mining and Technology, 221116 Xuzhou, China

## Abstract

*In-situ* processing of tungsten aluminide and tungsten reinforced aluminium matrix composites from elemental tungsten (W) and aluminium (Al) was investigated by thermal analysis and pulsed current processing (PCP). The formation mechanism of tungsten aluminides in 80 at.% Al-20 at.% W system was controlled by atomic diffusion. The particle size of W and Al in the starting powder mixture regulated the phase formation and microstructure. PCP of micron sized elemental Al and W resulted in formation of particulate reinforcements, W, Al_4_W and Al_12_W, dispersed in Al matrix. W particles were surrounded by a ~3 μm thick dual-layer structure of Al_12_W and Al_4_W. The hardness of Al matrix, containing Al_12_W reinforcements, was increased by 50% compared to pure Al, from 0.3 GPa to 0.45 GPa and W reinforcements showed a hardness of 4.35 GPa. On PCP of 80 at.% Al-20 at.% W mixture with particle size of W and Al ~70 nm, resulted in formation of Al_4_W as major phase along with small fractions of Al_5_W and unreacted W phase. This suggested strongly that the particle size of the starting elemental Al and W could be the controlling parameter in processing and tailoring of phase evolution, microstructure of particulate reinforced Al matrix composite.

## Introduction

Aluminium based metal matrix composites (Al-MMCs) are attractive for automotive and aerospace applications due to excellent combination of their physical and mechanical properties and oxidation resistance^1–3^. Reinforcement materials such as Al_2_O_3_, SiC, TiC are commonly utilized to fabricate particulate reinforced Al-MMCs^4–7^. The addition of particulates to Al matrix are aimed at dispersing particulate reinforcements homogeneously in Al matrix to obtain a homogenous microstructure and isotropic properties^8^. The reinforcements are incorporated either by *ex-situ* processing of metallic matrix and reinforcement where the reinforcements are synthesized prior to addition to Al matrix, or by *in-situ* processing of metallic matrix with constituent elements reacting to form particulate reinforcements during the fabrication process such as liquid metal infiltration^9^, powder metallurgy^10,11^, various casting techniques^12,13^ and mechanical alloying^14^. The *in-situ* processing offers several advantages over *ex-situ*, for instance, homogeneous distribution of reinforcements in matrix phase, clean interface between matrix and reinforcements, superior properties and high energy efficiency^15–17^.

Incorporation of Al based intermetallic compounds as reinforcement to Al matrix has attracted interest and showed promising improvements in properties of Al-MMCs due to formation of strong interfacial bonds between the intermetallic compound and Al matrix^18^. The formation mechanism of intermetallic compound during *in-situ* processing can be explained by diffusion process^19^ or thermal explosion (TE)^20^. Sun *et al*.^21^ reported that Young’s modulus of fabricated Al/Al_3_Ti composite with 9.4 mol. % Ti was improved to 110 GPa, which was about 57% higher than that of pure Al, 70 GPa. NiAl reinforced Nickel matrix composite fabricated by Mizuuchi *et al*.^22^ showed an increased tensile strength of 500 MPa compared to pure Ni (about 200 MPa).

The addition of high strength refractory materials into a ductile matrix has also been reported to improve the mechanical properties of the composite^23,24^. This study incorporates tungsten (W) to fabricate Al-MMCs, aiming for the reinforcing effect from both W-Al intermetallics and particulate W. W is known to have high melting point, high strength, low coefficient of thermal expansion. The equilibrium Al-W phase diagram shows that three intermetallic compounds can stably exist at room temperature, Al_4_W, Al_5_W and Al_12_W. Ideally, the addition of particulate W and W-Al intermetallics in Al matrix can improve the mechanical performance such as specific strength, oxidation resistance and thermal stability over the elemental Al and W. However, the low solubility of W in Al and high reactivity of W with Al raises the difficulty to fabricate Al-W composites by equilibrium synthesis routes. Y.C. Feng *et al*. have synthesized Al_12_W reinforced Al-MMC from Al99W1 (vol.%) system through reaction sintering followed by hot extrusion with small volume fraction of reinforcements^25^. The final composite consists of Al_12_W particles with a size of <1 µm distributed in the Al matrix after the complex sintering process. Mechanical alloying has been used to form Al-W composites^26,27^, but it involves long processing time (normally >10 h) of high-energy ball milling and has poor control of the alloying process. High sintering temperature and prolonged sintering time lead to heterogeneous composite structure^28,29^ and unexpected grain growth. In this regard, pulsed current processing (PCP), as a novel and non-equilibrium sintering technique, has shown its advantage in fabricating Al-MMCs from consolidating powdered metals at relative low temperature and within a short time^30,31^. PCP combines uniaxial force, pulsed direct electrical current and low atmosphere pressure during sintering, which gives fully dense materials^32^. Here, we report that the atomic diffusion kinetics in Al-W system in combination with non-equilibrium processing resulted in fabrication of a W and W-Al intermetallics reinforced Al matrix composite. The composite showed remarkable densification during processing and uniform distribution of particulate reinforcement in the Al matrix. Two significant factors, heating rate and sintering time that control the phase formation and microstructure evolution of Al-W composites were investigated by thermal analysis. The effect of particle size, i.e. diffusion length scale would be discussed.

## Results and Discussion

**Figure 1:**
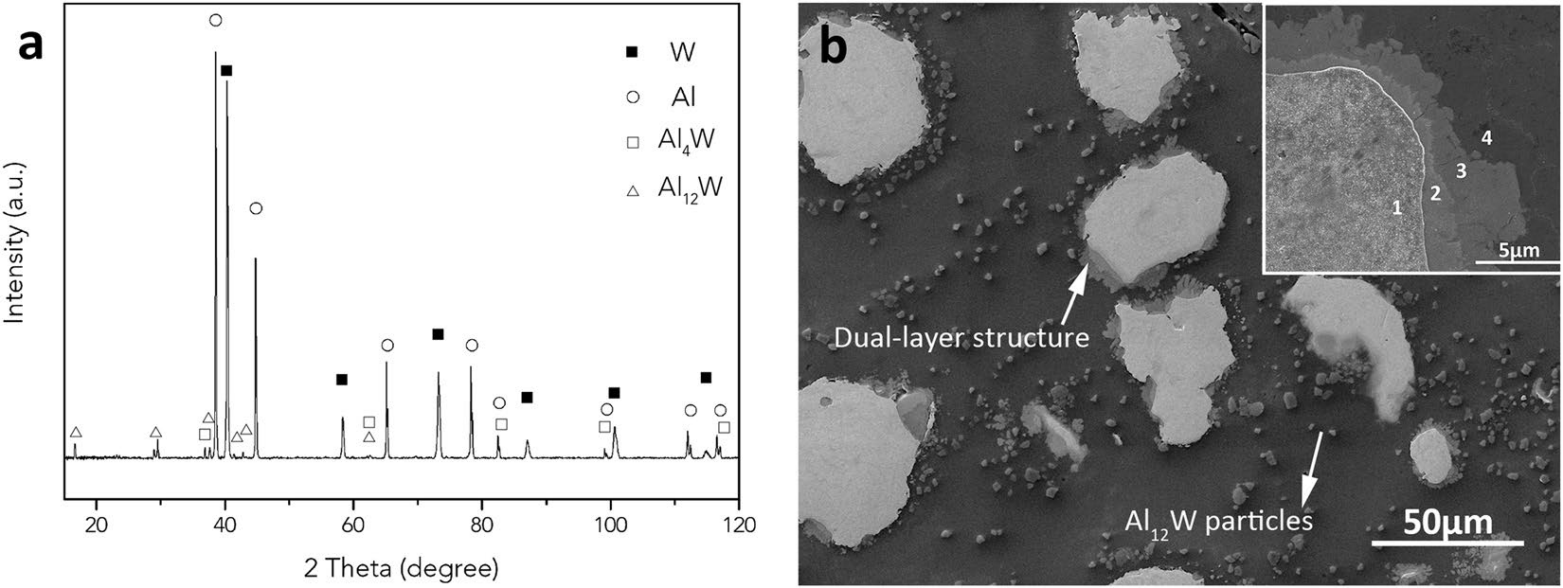
The microstructures and composition identification of m-80Al20W PCP sintered Al-MMC. (**a**) XRD pattern reveals the formation of Al_4_W and Al_12_W; (**b**) the reinforcements are uniformly distributed in Al matrix. The reinforcement phases 1→4 present in the inset are: W, Al_4_W, Al_12_W and Al.

The pulsed current processing (PCP) of micron-sized Al (80 at.%) and W (20 at.%) powder mixture (denoted as m-80Al20W) resulted in formation of particulate reinforced Al matrix composite (Al-MMC). The density of the composite is 6.3 g/cm^3^, which is higher than its theoretical density of 5.9 g/cm^3^, calculated using the rule of mixture. The fact that the density of the composite has exceeded theoretical density could be attributed to the loss of Al from the die during PCP under combined effect of temperature, pressure and vacuum. The X-ray diffraction (XRD) data of Al-MMC in Fig. 1a indicates the formation of Al_4_W and Al_12_W intermetallic phases in addition to Al matrix and W reinforcements. This infers that Al and W reacted during PCP to form equilibrium intermetallic phases following the Al-W equilibrium phase diagram. The microstructure of Al-MMC in Fig. 1b shows the homogenous distribution of bimodal particulate reinforcements of 2–5 µm (Al_12_W) and 20–50 µm (multi-phase structure) in Al matrix. The XRD data and microstructure in Fig. 1 suggest that the Al-MMC consist of Al and W as major phases and small fraction of Al_4_W and Al_12_W intermetallics. The high magnification inset in Fig. 1b reveals the formation of intermetallic dual-layer structure at the Al/W interface. The energy dispersive analysis (EDS) confirms that layers formed around W particles are Al_4_W and Al_12_W intermetallics (Supplementary information, Fig. S1). The intermetallic phases grow in layers as suggested by multilayer growth mechanism in diffusion couples^19,33,34^. Initial nucleation of intermetallic compounds starts at the boundary of Al/W interface. The subsequent grain growth is controlled by solid-solid diffusion. Because the Al_12_W phase is relatively rich in Al, it expands predominately towards Al phase, while Al_4_W grows towards W. Meanwhile, the outmost Al-Al_12_W interface shows evidence of disintegration and migration of particles from Al_12_W layers resulting in observed presence of Al_12_W particle reinforcements of 2–5 μm in Al matrix (Fig. 1b). The W reinforcements showed a hardness of elemental W, 4.35 GPa, and Al matrix reinforced with Al_12_W particles showed a hardness of 0.45 GPa, which was increased 50% of pure Al due to the incorporation of intermetallic phases in Al matrix.

**Figure 2:**
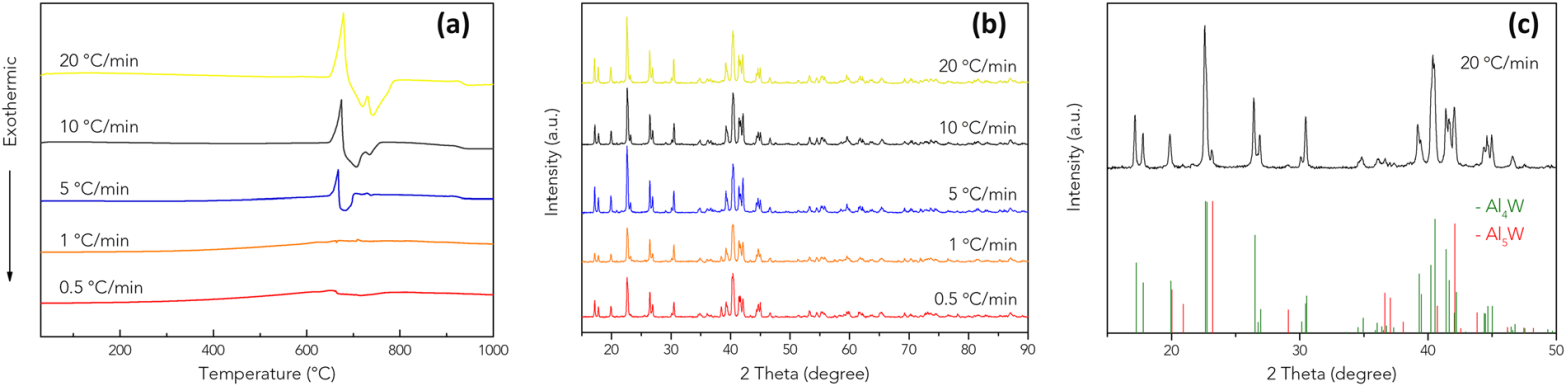
(**a**) DSC data, (**b**) XRD patterns obtained from m-80Al20W green compacts sintered to 1000 °C with different heating rates. (**c**) Al_4_W and Al_5_W reference patterns are inserted (ICSD standard pattern No. 01-072-5022 and 03-065-4779, respectively**)** for the comparison with the XRD pattern obtained from 20 °C/min DSC experiment.

The influence of heating rate on inter atomic diffusion process in m-80Al20W system and formation of intermetallic compounds in the composite was studied by Differential Scanning Calorimeter (DSC) in the temperature range 30–1000 °C. DSC curves of 5 °C/min, 10 °C/min and 20 °C/min in Fig. 2 show distinguished thermal events, indicating higher data resolution at raised heating rate^35,36^. The onset of first endothermic event that starts at around 660 °C is attributed to the melting of Al, as Al has a melting point of 660 °C. The melting of Al is followed immediately by the formation of intermetallics which is related to the exothermic peaks. In the case of heating rate of 20 °C/min, the two exotherms appear at 720 °C and 738 °C respectively, which refer to the formation of two different compounds: Al_4_W and Al_5_W. The XRD patterns in Fig. 2(c) show good consistency with Al_4_W and Al_5_W reference diffraction patterns indicating a high fraction of equilibrium intermetallic phase Al_4_W was formed and low fraction of Al_5_W was also detected (Supplementary Information, Fig. S2), which is consistent with the appearance of two exothermic peaks in DSC data (Fig. 2(a)). The complete phase transformation is attributed to lower heating rate compared to PCP.

**Figure 3:**
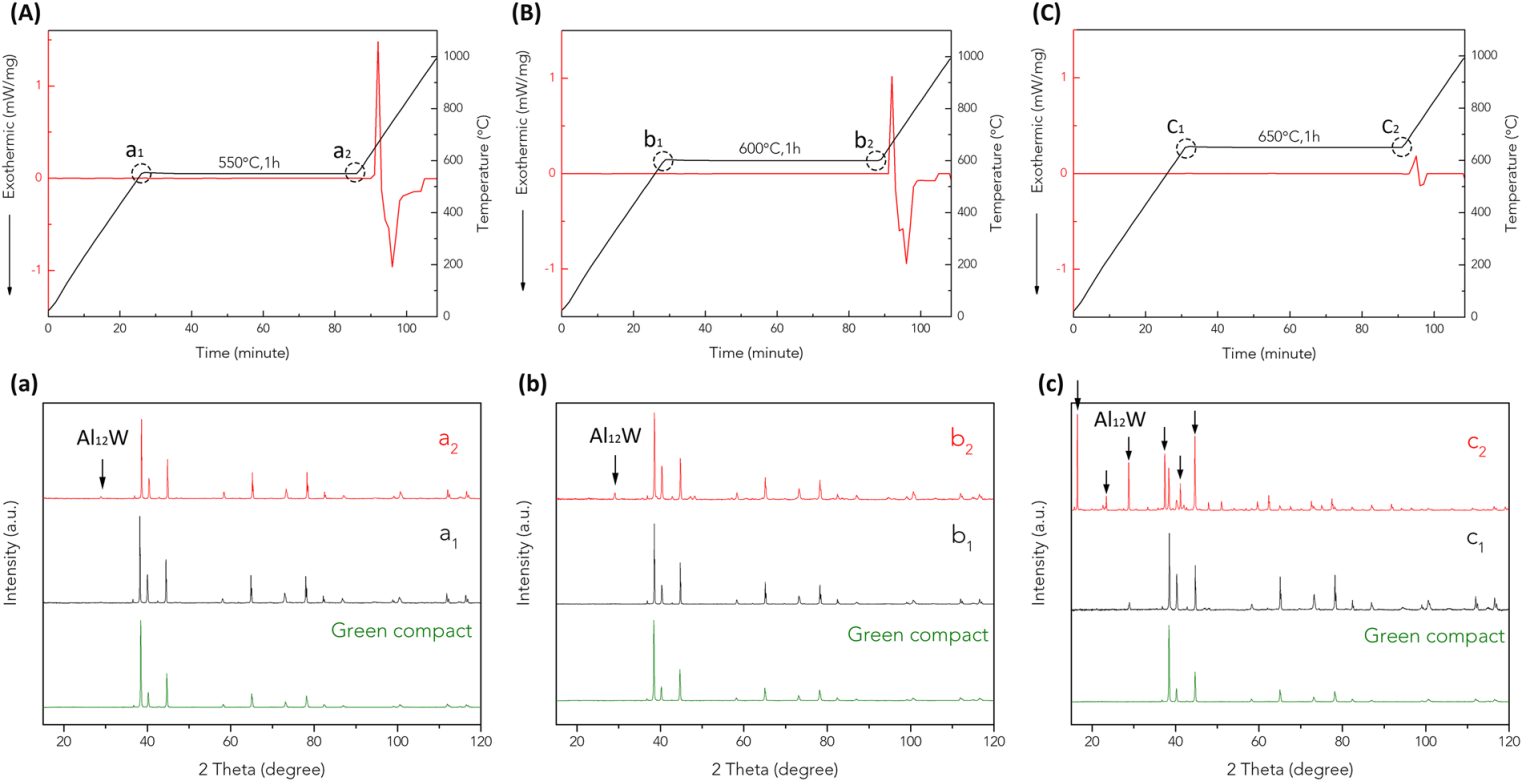
DSC curves and XRD patterns of m-80Al20W green compacts sintered to 1000 °C. The temperature was held at (**A**) 550 °C, (**B**) 600 °C and (**C**) 650 °C for 1 hour, respectively. Baselines were subtracted by Origin. (**a**), (**b**) and (**c**) are XRD patterns for m-80Al20W compacts that are treated with different temperature profiles in corresponded thermal programs A, B and C.

In order to elucidate the phase evolution in Al-W system in solid state, m-80Al20W compacts were heated to temperatures below the melting point of Al and held for 1 hour. The artifacts of DSC curves caused at step change on the onset and offset of temperature holding platforms were removed by adjusting baselines in the software Origin. As shown in Fig. 3A–C, the first obvious exothermic event showed up when Al melts at around 660 °C in all three DSC curves. However, the intensity of endothermic melting peaks and following exothermic peaks become less pronounced as the holding temperature increases from 550 to 650 °C. The tendency of reduction in reaction enthalpy implies that the Al and W diffuse to form intermetallics before Al melts. The amount of reacted elemental Al increases with the increasing holding temperature as higher temperature promotes atomic diffusion^37^, leading to the formation of higher contents of intermetallic compounds. Similar diffusion-controlled mechanism of intermetallic formation in Au-Al system was reported by Elliott Philofsky^19^. In their study, different Au-Al intermetallic compounds were synthesized at temperature range 200–460 °C based on diffusion of solid-state metals. Liang *et al*. reported the formation of TiAl_3_ at 600 °C by solid state reaction^38^.

Based on the experiments with the holding temperature platforms, the diffusion-controlled mechanism is further verified by terminating the heat treatment at different steps: the onset and the offset of the 1 hour holding platform (Al-W compacts at a_1_, a_2_, b_1_, b_2_, c_1_ and c_2_ in Fig. 3A–C) and determining the phase evolution by XRD. According to the analysis results of XRD patterns in Fig. 3a–c, Al-W compact at a_1_ consists of Al and W, which implies that no reaction has taken place at 550 °C. At a_2_, XRD peak of intermetallic compound Al_12_W is detected in Al-W compact (Fig. 3a), which is treated at 550 °C and held for 1 hour. Compare with the PCPed m-80Al20W, where the diffusion process was accelerated by the combined effect of rapid heating rate, current and uniaxial force resulting in formation Al_4_W and Al_12_W intermetallic compounds, the XRD data at a_2_ suggests that Al_12_W forms first in Al-W system. On increasing the holding temperature to 600 °C, the formation of intermetallic is promoted in Al-W compact b_2_. The XRD data of Al-W compact at b_2_ shows increase in diffraction intensities of Al_12_W at b_1_ at 2θ = 28.8° in Fig. 3b. On increasing the temperature to 650 °C, the XRD data of Al-W compact at c_2_ shows the high intensity Al_12_W peaks compared to a_2_ and b_2_ (Fig. 3c). In addition to Al_12_W peaks, the Al-W compact at c_2_ shows the presence of Al_4_W (See Fig. S3 in Supplementary Information). It indicates that higher temperature and longer holding times are required to obtain equilibrium intermetallic phase Al_4_W in m-80Al20Wsystem.

**Figure 4:**
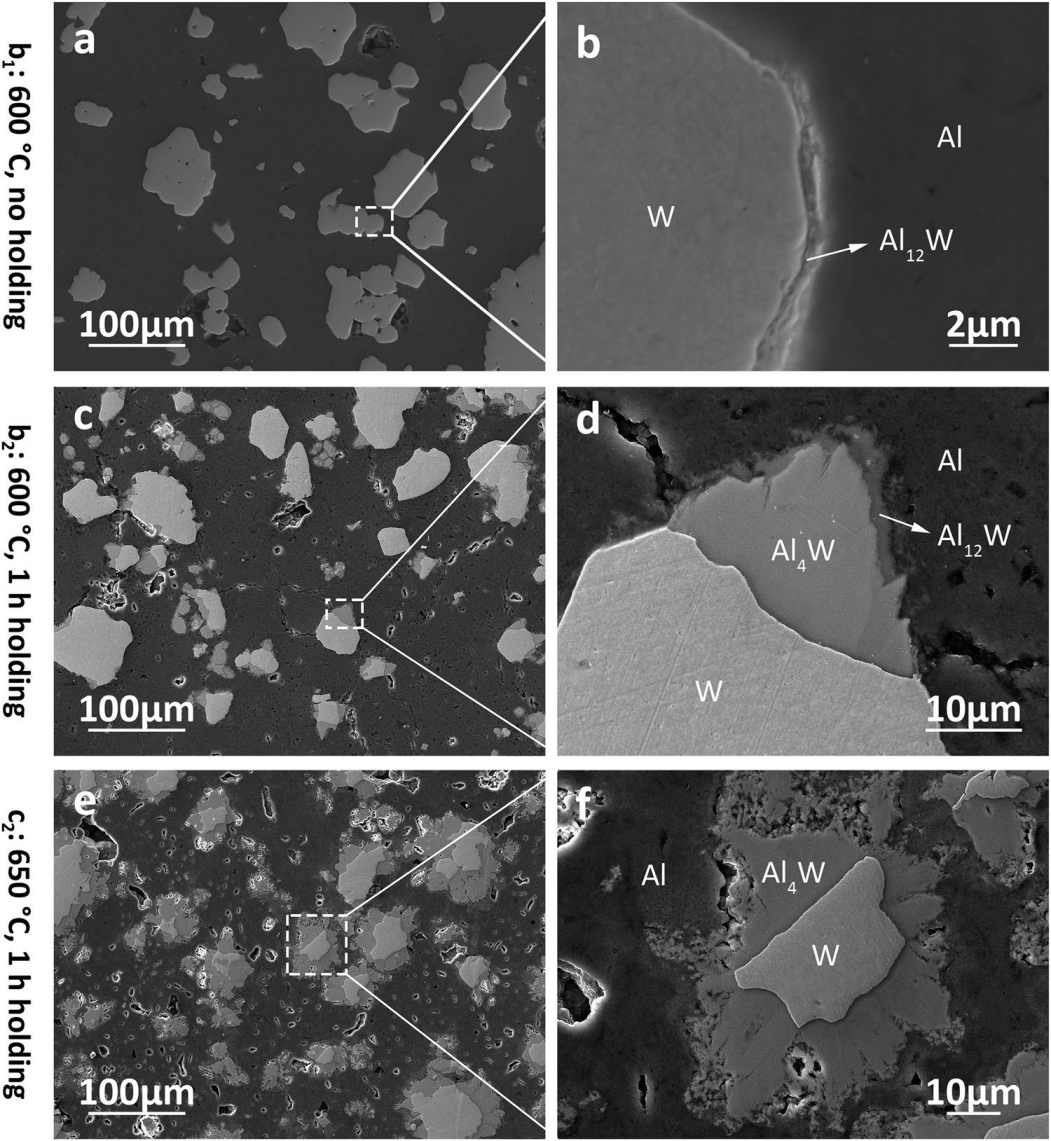
Microstructure of m-80Al20W compacts heated with different temperature profiles (b_1_, b_2_ and c_2_). The high magnification images (**b**), (**d**) and (**f**) show that increased temperature and prolonged heating promote the phase transformation.

The microstructure of Al-W compacts at b_1_, b_2_ and c_2_ are presented in Fig. 4. A thin layer (approximately 500 nm) is found between Al and W phase at b_1_ (Fig. 4a and b) indicating the formation of initial diffusion zone at the boundaries of W particles at 600 °C. The composition of the 500 nm layer is identified as intermediate phase Al_12_W according to the XRD data. The formation of Al_12_W is controlled by diffusion of Al and W atoms and subsequent nucleation and growth from this diffusion zone. According to Kirkendall effect, the atomic diffusion involves the exchange between atoms and vacancies. Therefore, the directional mass flow from one phase towards another that is caused by diffusion process should be balanced by opposite directional vacancy flow^39^. The increase in porosity in Al matrix (Fig. 4a,c and e) may result from the directional mass flow from Al to W, i.e. Al diffuses faster than W in Al-W system, with increase in the volume fraction of intermetallic phases. Massive diffusion of Al atoms into the diffusion zone results in formation of intermetallic compound with high atomic fraction of Al, i.e. Al_12_W in Fig. 4b. The growth of intermetallic phases (≈10 µm thickness) results during 1 hour of extended holding time at 600 °C as shown in Fig. 4c and d. EDS spectra of m-80Al20W compact at b_2_ (Supplementary Information, Fig. S4) reveals that the formation of Al_4_W between W particle and Al_12_W. As the initial solid state reaction between Al and W formed Al_12_W at Al/W interfaces, the following reaction is controlled by the diffusion of atoms at the W/Al_12_W interface, so Al_4_W is formed in between Al_12_W and W, and expected to continue until Al_12_W is consumed. Similar microstructure evolution and mechanism was reported in Al-Ti system^39,40^. Moreover, Fig. 4f shows that for the m-80Al20W compact at c_2_, the W particulates are surrounded by 10–20 µm Al_4_W layers (Supplementary Information, Fig. S5) by increasing the holding temperature from 600 °C to 650 °C. The microstructure evolution shown by the comparison among Fig. 4b,d and f is in accordance with XRD results in Fig. 3 confirming that the formation of W-Al intermetallics is controlled by solid-solid diffusion. Furthermore, it can be concluded that long holding time at high temperature are required to form equilibrium intermetallic phases in diffusion controlled phase transformation in m-80Al20W system. Therefore, the Al-W composites could be fabricated by tailoring the temperature, holding time and partial diffusion process.

It is suggested that in Al based binary system such as Fe/Al and Ni/Al, low solid solubility limits the diffusivity. The diffusion coefficient D of Fe and Ni in Al are presented by Ken-ichi Hirano^41^ as: ${D}_{{Fe}/{Al}}\,=\,4.1\times {10}^{-9}\exp (-\frac{13900}{{RT}})$ and ${D}_{{Ni}/{Al}}\,=\,2.9\times {10}^{-8}\exp (-\frac{15700}{{RT}})$, where R and T represent the universal gas constant and temperature, respectively. The values of pre-exponential diffusion coefficient D_0_ is extremely small compared to the self-diffusion coefficient of Al^42^. Similarly, W should also have low diffusivity in Al because of low solid solubility of W in Al (about 0.45 at.% at 600 °C). According to Fick’s first law^43^, the diffusion flux is proportional to diffusivity while inversely proportional to diffusion distance. The maximum diffusion distance in Al-W system is defined by particle size. Therefore, reduced particle size should theoretically improve the diffusion controlled phase transformation. In this regard, the starting material was replaced by nano-sized 80Al20W powder (n-80Al20W) and treated with the same PCP route as m-80Al20W.

**Figure 5:**
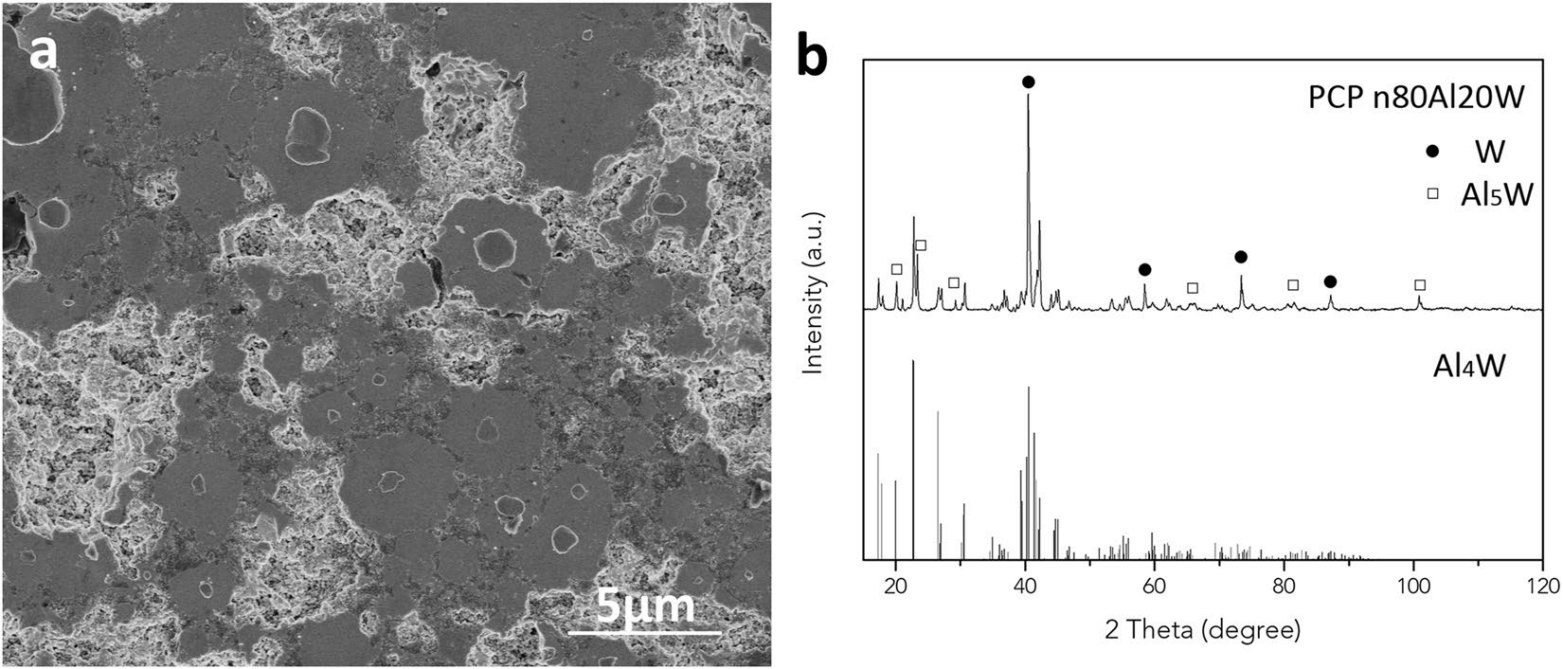
The microstructure and composition identification of PCPed n-80Al20W sample. (**a**) the microstructure shows small amount of unreacted W and high porosity in the composite; (**b**) the XRD pattern of the PCPed n-80Al20Wsample and inserted Al_4_W reference pattern (ICSD standard pattern No. 01-072-5022) indicates the composite consist of Al_4_W as the main product and small amount of Al_5_W and W.

**Figure 6:**
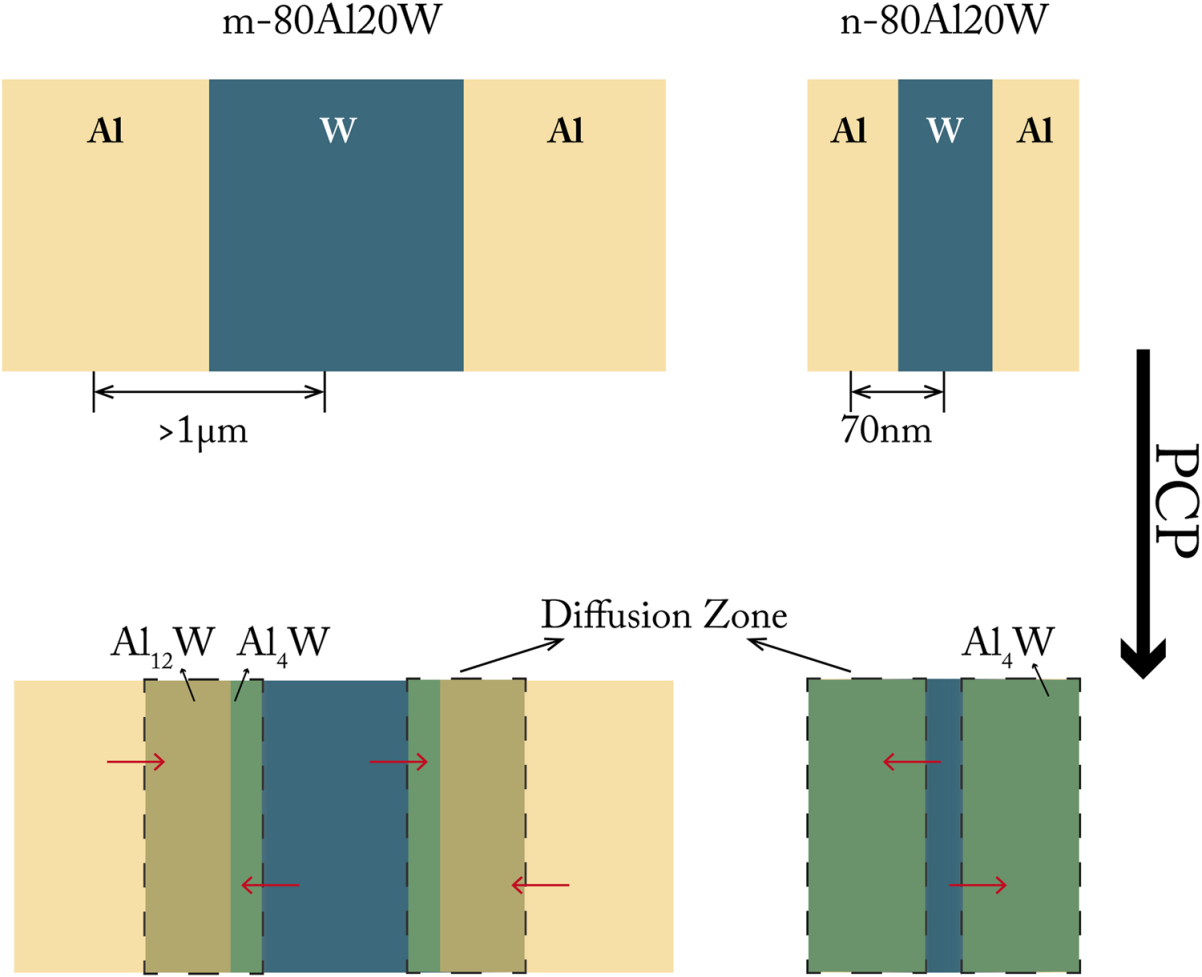
Schematic of the diffusion controlled mechanism in Al-W system as a function of particle size, i.e. diffusion path. By using nano sized particles, the diffusion path is reduced down to 70 nm, which consequently resulted in formation of more equilibrium phase Al_4_W in the composite (minor amount of Al_5_W is not shown here).

The PCPed n-80Al20W resulted in formation of intermetallic compounds Al_4_W as the dominating phase and Al_5_W, along with trace amount of unreacted W in the sample (Fig. 5). The sample showed a hardness of 1.55 GPa. M.G. Golkovski *et al*.^44^ reported the nanoharness of Al_4_W to be 9–11 GPa. The reason of PCPed n-80Al20W exhibits lower hardness than pure Al_4_W is attributed to the high porosity of the sample^45^. The calculated crystallite size of Al_4_W is 65.9 nm, calculated by Sherrer’s equation. High porosity in the PCP compact is revealed in Fig. 5a. The lack of densification arises from the reduced diffusion distance as well as increased contact area between Al and W particles, which allowed massive diffusion of the atoms and vacancies during the diffusion process. Voids are therefore created in the original position of Al and W atoms according to Kirkendall effect^46,47^. As described in Fig. 6, in order to achieve complete phase transformation, the distance that Al and W atoms need to transport and form Al-W intermetallics equals to the diameter of the starting particle diameter. The usage of nano sized particles reduced the diffusion path from >1 µm down to 70 nm, thus leading to equilibrium phase transformation of the raw materials. The thorough phase transformation indicates that, despite of the barrier of phase transformation that is posed by limited diffusivity, the diffusion process in n-80Al20W system is greatly enhanced compare to m-80Al20W with reduced diffusion distance, i.e. particle size in PCP.

In conclusion, a particulate tungsten aluminides/W reinforced Al-MMC has been successfully fabricated by *in-situ* PCP. The Al-MMC contains uniformly distributed W particulates with Al_4_W and Al_12_W dual-layer structure in Al matrix. The mechanical property of the composite fabricated from m-80Al20W is improved with a hardness of 0.45 GPa in Al matrix and 4.35 GPa for W reinforcements. We have shown that the formation mechanism of W-Al intermetallic compounds is controlled primarily by atomic diffusion process between solid state Al and W. Shorter diffusion path, i.e. smaller starting particle size showed strong effect on the microstructure evolution and phase formation with phase transformation toward equilibrium intermetallic phase Al_4_W. The temperature and time barrier that arise from limited solid state diffusion rate was overcome. The results presented in this work provide a guideline for designing fabrication routes for intermetallic reinforced Al-MMCs.

## Methods

### Starting materials

Elemental aluminium (APS 1–15 µm, 99.5% purity, Alfa Aesar) and tungsten (<1 µm, 99.95% purity, Alfa Aesar) powders were used as reactant micron-sized powders. Meanwhile, aluminium (70 nm, 99.9% purity, US Research Nanomaterials, Inc.) and tungsten (70 nm, 99.9% purity, US Research Nanomaterials, Inc.) were selected to prepare nano-sized powder precursors. Both micron- and nano-sized powders (denoted as m-80Al20W and n-80Al20W) were prepared by mixing 80 at.% Al − 20 at.% W powders. The elemental powder mixtures were thoroughly mixed in a ball mill for 2 h using 4 mm stainless steel balls at ambient temperature. The weight ratio of ball to powders was 5:1. The environment for ball milling was air and argon for m-80Al20W and n-80Al20W, respectively.

### Processing

Pulsed current processing (PCP) for both m-80Al20W and n-80Al20W were conducted on Dr. Sinter 2050 (Sumitomo Coal Mining Co., Ltd., Japan). The samples were sintered in vacuum by following the same PCP thermal program: the powder was filled into 12 mm graphite die and heated to 550 °C with a heating rate of 100 °C/min, and held for 2 minutes. The pressure employed during sintering was 20 MPa.

The m-80Al20W powder mixture were consolidated into green compacts by a laboratory hydraulic press (Manual Hydraulic Press – 15Ton, Specac, England) at a pressure of 5 Ton before performing thermal analysis in Differential Scanning Calorimeter (STA 449 C Jupiter®, NETZSCH, Germany). The effect of different heating rates was done by performing the thermal program from 30 °C to 1000 °C with different heating rates (0.5, 1, 5, 10 and 20 °C/min, respectively). In order to study the effect of prolonged holding time, the m-80Al20W compacts were heated from 30 °C to 1000 °C with a heating rate of 20 °C/min, but with different holding temperature stages (550 °C, 600 °C, and 650 °C) for 1 hour during the process.

### Characterization

A Matsuzawa MXT-CX microhardness tester (Matsuzawa Co., Japan) was used to measure the hardness of Al-MMC sample fabricated from m-80Al20W in PCP. The applied load for microhardness testing was 50 g. Each hardness value was calculated from the average of 9 indentations. The sintered density was measured by Archimedes water immersion method. The theoretical density was calculated based on the mixture rule at the assumption of no reaction taking place.

Both the PCP samples and the samples from thermal analysis were characterized by an X-ray diffractometer (Empyrean, PANalytical, United Kingdom) and Cu-Kα radiation with a wavelength of 0.154 nm. The samples was cleaned and polished on the analysed surface before sent to XRD characterization. The XRD patterns were analysed by the software PANalytical X’Pert HighScore Plus to determine the compositions. Thermal analysis was performed using Netzsch TA software.

All the samples were prepared by following standard metallographic procedures for microstructure characterization. Because of different requirement of the image resolution, Scanning Electron Microscopy JSM- IT300 (JEOL, Tokyo, Japan) mounted with an energy dispersive spectrometer (EDS) that is calibrated with Cobalt standard, and Magellan 400 XHR-SEM (FEI Company, Eindhoven, the Netherlands) were used for microstructure observation for samples after thermal analysis and PCP samples.

## Electronic supplementary material

Supplementary Information
