# Supplementary material for: Aluminium matrix tungsten aluminide and tungsten reinforced composites by solid-state diffusion mechanism
Source: Sci Rep. 2017 Sep 28;7:12391. doi: 10.1038/s41598-017-12302-w (PMC5620082; doi:10.1038/s41598-017-12302-w)
Supplement: Supplementary file 1 — Supplementary Information [file 41598_2017_12302_MOESM1_ESM.pdf]

Supplementary information for:

Aluminium matrix tungsten aluminide and tungsten reinforced composites by solid-state diffusion mechanism

Hanzhu Zhang[1], **Peizhong Feng**[2], **Farid Akhtar**[1,*]

[1]Division of Materials Science, Luleå University of Technology, 971 87 Luleå, Sweden

[2]School of Materials Science and Engineering, China University of Mining and Technology, 221116 Xuzhou, China

[*]farid.akhtar@ltu.se

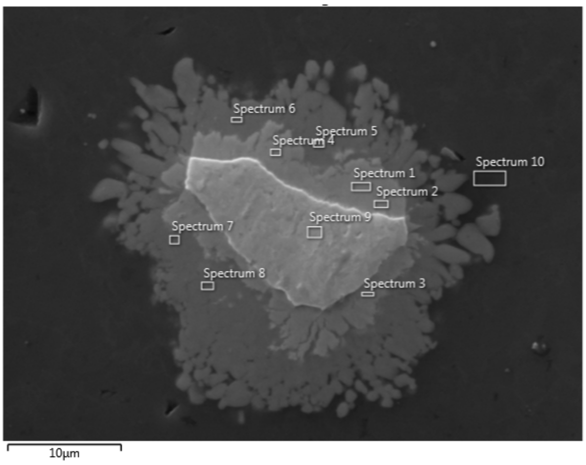

Spectra number	Al (at.%)	W (at.%)
1	78.9	21.1
2	76.8	23.2
3	77.9	22.1
4	78.5	21.5
5	91.2	8.8
6	91.9	8.1
7	90.4	9.6
8	91.4	8.6
9	0	100
10	100	0

Figure S1. The energy dispersive analysis (EDS) of PCPed m-80Al20W compact. The phases of the dual-layer structure formed around W particles are identified as Al_4W (spectra 1-4) and $Al_{12}W$ (spectra 5-8) intermetallics.

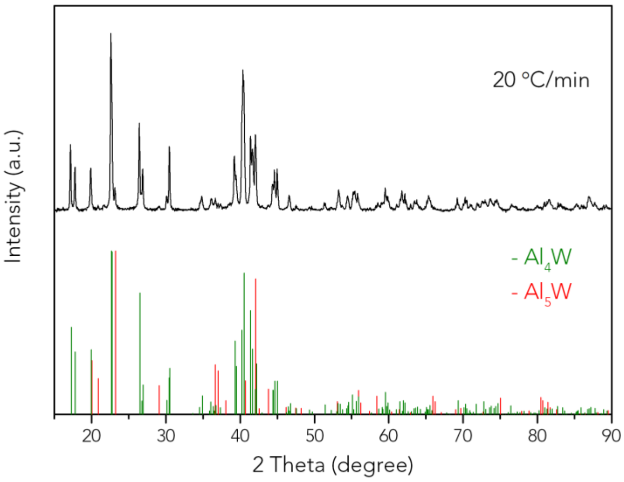

Figure S2. XRD pattern of m-80Al20W sintered to 1000 °C at 20 °C/min and XRD reference patterns of Al_4W and Al_5W (ICSD standard pattern No. 01-072-5022 and 03-065-4779, respectively).

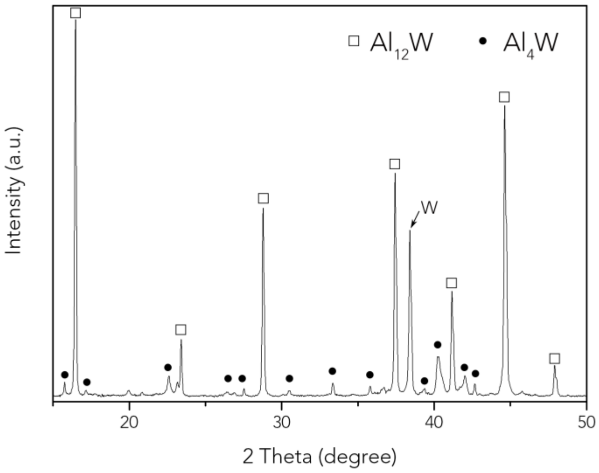

Figure S3. XRD pattern of m-80Al20W green compact sintered to 1000 °C with holding at 650 °C for 1h (sample c_2). The XRD pattern is shown at 2θ=15-50°, indicating the formation of two intermetallic compounds Al_4W and $Al_{12}W$.

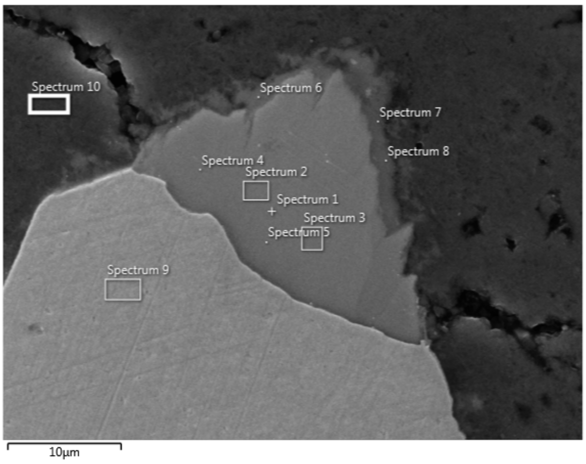

Spectra number	Al (at.%)	W (at.%)
1	81.2	18.8
2	80.8	19.2
3	80.2	19.8
4	81.3	18.7
5	81.5	18.5
6	92.2	7.8
7	94.3	5.7
8	94.5	5.5
9	0	100
10	100	0

Figure S4. The energy dispersive analysis (EDS) of m-80Al20W compact at b_2 (600°C for 1hour). The intermetallic compounds formed at the W/Al interface are identified as Al_4W (spectra 1-5) and $Al_{12}W$ (spectra 6-8) intermetallics.

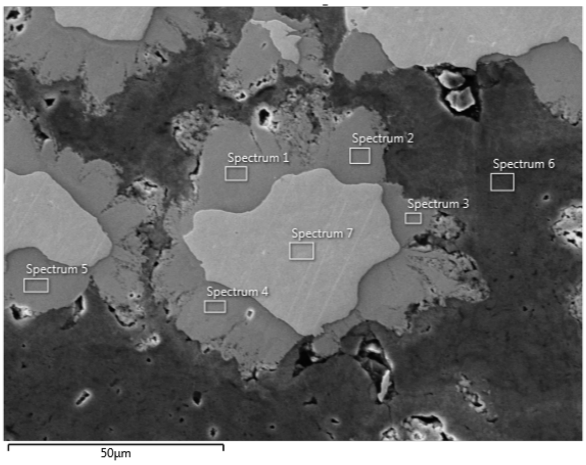

Spectra number	Al (at.%)	W (at.%)
1	77.7	22.3
2	78.1	21.9
3	79.2	20.8
4	78.1	21.9
5	81.5	18.5
6	100	0
7	0	100

Figure S5. The energy dispersive analysis (EDS) of m-80Al20W compact at c_2 (650°C for 1hour). The intermetallic phase formed at the W/Al interface is identified as Al_4W (spectra 1-5).
